# The predictive value of T-tau and AB1-42 levels in idiopathic normal pressure hydrocephalus

**DOI:** 10.1007/s00701-017-3314-x

**Published:** 2017-09-09

**Authors:** Claudia L. Craven, Irene Baudracco, Henrik Zetterberg, Michael P. T. Lunn, Miles D. Chapman, Neghat Lakdawala, Laurence D. Watkins, Ahmed K. Toma

**Affiliations:** 1 Victor Horsley Department of Neurosurgery, National Hospital for Neurology and Neurosurgery, Queen Square, London, WC1N 3BG UK; 2 Department of Molecular Neuroscience, UCL Institute of Neurology, Queen Square, London, WC1N 3BG UK; 3 Department of Psychiatry and Neurochemistry, Institute of Neuroscience and Physiology, The Sahlgrenska Academy at the University of Gothenburg, Mölndal, Sweden; 4 Department of Neurology, National Hospital for Neurology and Neurosurgery, Queen Square, London, WC1N 3BG UK; 5 Department of Neuroimmunology, National Hospital for Neurology and Neurosurgery, Queen Square, London, WC1N 3BG UK

**Keywords:** AB1-42, Cerebrospinal fluid (CSF), Idiopathic normal pressure hydrocephalus (INPH), Neurodegenerative markers, T-tau

## Abstract

**Background:**

Idiopathic normal pressure hydrocephalus (INPH) has no reliable biomarker to assist in the selection of patients who could benefit from ventriculo-peritoneal (VP) shunt insertion. The neurodegenerative markers T-tau and Aβ1-42 have been found to successfully differentiate between Alzheimer’s disease (AD) and INPH and therefore are candidate biomarkers for prognosis and shunt response in INPH. The aim of this study was to test the predictive value of cerebrospinal fluid (CSF) T-tau and Aβ1-42 for shunt responsiveness. In particular, we pay attention to the subset of INPH patients with raised T-tau, who are often expected to be poor surgical candidates.

**Methods:**

Single-centre retrospective analysis of probable INPH patients with CSF samples collected from 2006 to 2016. Index test: CSF levels of T-tau and Aβ1-42. Reference standard: postoperative outcome. ROC analysis assessed the predictive value.

**Results:**

A total of 144 CSF samples from INPH patients were analysed. Lumbar T-tau was a good predictor of post-operative mobility (AUROC 0.80). The majority of patients with a co-existing neurodegenerative disease responded well, including those with high T-tau levels.

**Conclusion:**

INPH patients tended to exhibit low levels of CSF T-tau, and this can be a good predictor outcome. However levels are highly variable between individuals. Raised T-tau and being shunt-responsive are not mutually exclusive, and such patients ought not necessarily be excluded from having a VP shunt. A combined panel of markers may be a more specific method for aiding selection of patients for VP shunt insertion. This is the most comprehensive presentation of CSF samples from INPH patients to date, thus providing further reference values to the current literature.

## Introduction

Idiopathic normal-pressure hydrocephalus (INPH) is a condition that predominantly affects the elderly population, has a prevalence of 0.02%–5.9% and affects an estimated 2 million people within Europe [6, 10]. INPH presents with a triad of cognitive deficits, impaired mobility and incontinence. The mainstay of treatment is cerebrospinal fluid (CSF) diversion via a ventriculo-peritoneal (VP) shunt [1, 3]. Selection of patients for VP shunt insertion remains a challenge since the triad of symptoms in INPH is common in the elderly population. The surgery for a VP shunt is not without risk and a proportion of those with INPH do not benefit [16, 18]. Whilst prognostic tests are becoming more accurate, INPH still has no reliable biomarker to assist in the selection of patients for a VP shunt or for the monitoring of shunt function [7, 9, 15, 16, 18].

Tau and Aβ1-42 are well defined as CSF biomarkers that aid in the diagnosis of Alzheimer’s disease (AD) and are potential prognostic markers in INPH [4, 8, 11–14]. Tau is a protein that promotes microtubule assembly and stability and is found in neuronal axons [4]. Aβ1-42 is a product of amyloid precursor protein (APP) that rapidly aggregates to form the main component of diffuse plaques [4]. The high CSF total-tau (T-tau) concentration is thought to reflect neuroaxonal degeneration/injury and low CSF Aβ1-42 correlates with senile plaque pathology [4, 8].

T-tau and Aβ1-42 levels have been found to successfully differentiate between AD and INPH [11, 12]. Patients with INPH consistently appear to have low CSF levels of T-tau and Aβ1-42 [8, 11–14]. A small subset of patients with INPH have raised T-tau levels on their initial lumbar samples, explained possibly by co-existing neurodegenerative disorder or a more progressive form of INPH [12]. It has been suggested that this subset of patients would be poor surgical candidates; however there is a paucity of literature describing outcomes in this group [12].

Over the last 10 years at this single centre, patients with probable INPH have had CSF samples analysed for total protein, T-tau and Aβ1-42 levels to investigate for co-existing neurodegenerative disease. Here we present the results of this large CSF sample cohort from INPH patients, including those with initial raised T-tau on their lumbar samples. We present the predictive values for lumbar and ventricular CSF T-tau and Aβ1-42 values for shunt responsiveness.

## Materials and method

### Study design

Single-centre retrospective analysis of probable INPH patients with CSF samples collected during August 2006 to January 2016. This study was reported in accordance with the STARD guidelines [5]. Clinical outcome (referenced standard), CSF samples analysis for T-tau and Aβ1-42 (index test) and radiological assessments were performed and recorded prospectively. The analysis of the data was performed retrospectively.

### Inclusion

Eligible patients required a diagnosis of probable INPH from a single centre. Only those with samples taken prior to VP shunt insertion (initial diagnostic LP or LD) or during insertion if a VP shunt were included into the predictive analysis. Formal consent for cerebrospinal fluid (CSF) sample analysis and use of results for research (including publication) was obtained.

### Exclusion

**Fig. 1:**
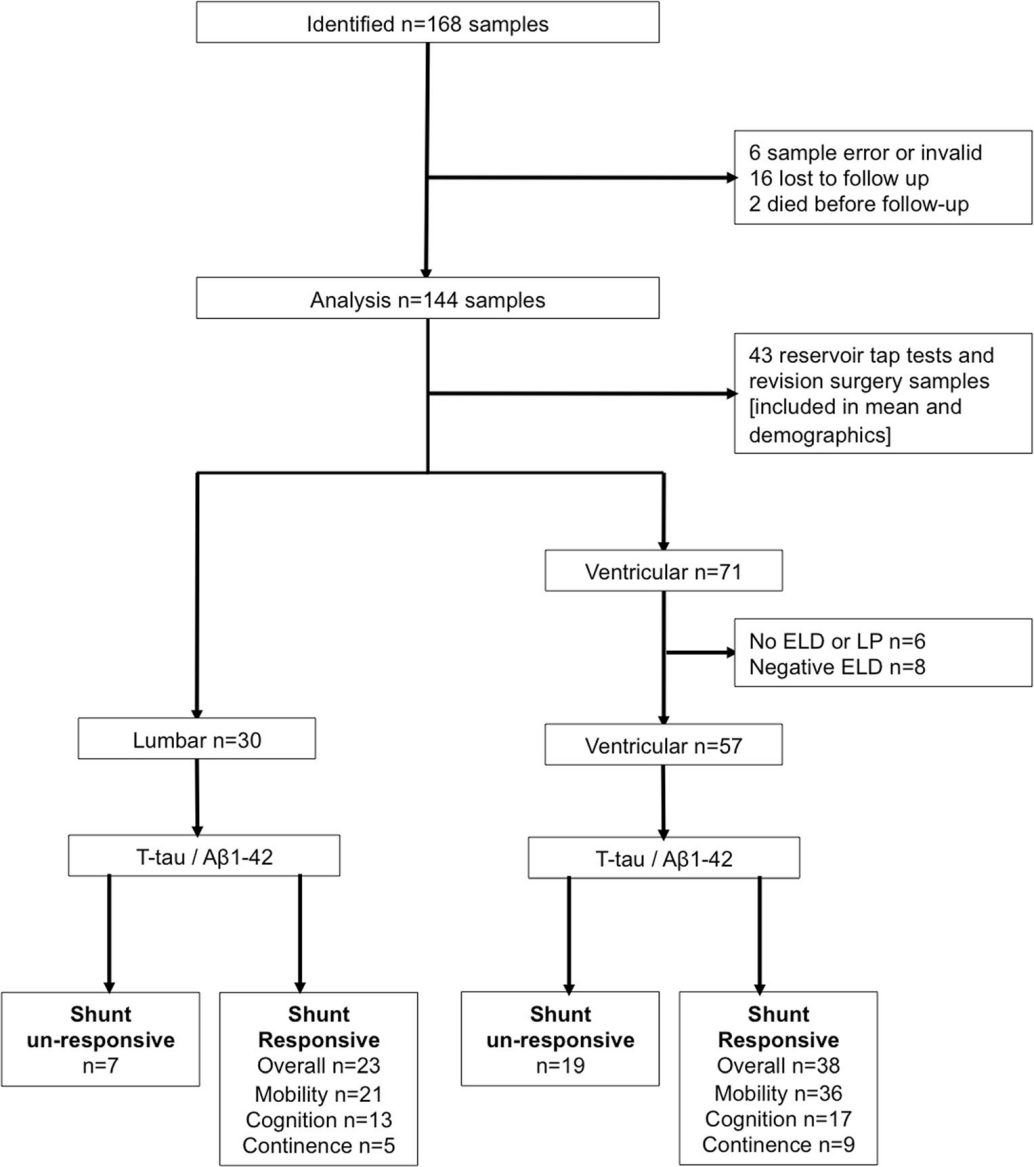
Flow chart of patient participants. A total of 87 samples, 71 primary ventricular and 30 primary lumbar (from 65 patients), were included in the final predictive value analysis. Secondary samples (43) and samples from those with poor or no ELD/LP were included in the longitudinal analysis of markers in CSF drainage and in analysis of rostro-caudal gradients

Exclusion criteria included loss to follow-up or death before follow-up. Those without prior lumbar drainage or lumbar puncture, or a poor response, were not fulfilling the international criteria for a diagnosis of probable INPH and therefore also not included in the ventricular predictive analysis (Fig. 1) [18]. These two groups were acknowledged in the demographic data and in the additional analysis.

### Diagnostic criteria

Three forms of evidence, based upon the international criteria for the diagnosis of probable INPH, were used to aid selection of patients for VP shunt insertion [18]: (1) patients over 40 years of age with a clinical insidious history of the typical triad of cognitive decline, mobility impairment and urinary incontinence, (2) characteristic brain imaging (MR images or CT) showing an un-obstructive ventriculomegaly and (3) positive clinical response in either mobility or cognition or a reduction in episodes of urinary incontinence after extended lumbar drainage (ELD) or lumbar puncture (LP). Patients were deemed to have a diagnosis of co-existing neurodegenerative disease if they had objective evidence (clinical, radiological and biochemical) and formal diagnosis made independently by a consultant neurologist.

### Index test: CSF analysis

CSF was sampled for T-tau and Aβ1-42 during any of the following interventions: LP, ELD or infusion study, VP shunt, VP shunt revision or reservoir tap test. Samples taken during the ELD protocol were taken at the time of drain or catheter insertion and not after a period of extended drainage. CSF samples were collected under sterile conditions into 10-ml Sartstedt polypropylene tubes. CSF sample collection and storage methods were all in accordance with the consensus guidelines for CSF biobanking [21]. Samples were sent for biochemical and enzyme-linked immunosorbent (ELISA) analysis to measure concentrations of T-tau (INNOTEST hTAU ELISA, Fujirebio, Ghent), Aβ-42 [INNOTEST β-amyloid (1–42), Fujirebio, Ghent] and total protein. A technician, blinded to the clinical results, prospectively recorded levels of T-tau and Aβ-42. Longitudinal stability in the measurements was ascertained using an elaborate programme of internal quality control (QC) samples. The laboratory also takes part in the Alzheimer’s Association external QC programme for CSF biomarkers [6]. Intra- and inter-assay coefficients of variation were 12 and 15% for Aβ1-42, respectively, and 3 and 12%, respectively, for T-tau.

### Reference standard: clinical outcome

The clinical outcome groups ‘shunt responsive’ or ‘shunt unresponsive’ provided the reference standard. Outcome measures were recorded prospectively and analysed retrospectively. Three main outcome objective measures were analysed: (1) Wechsler Adult Intelligence Scale R (WAIS-R) neuropsychology report (any improvement observed in verbal IQ, performance IQ or full scale IQ), (2) timed 10-m walking test (a minimum of 5% improvement in either time in seconds or number of steps, or both) and (3) bladder control (with improvement being the reduction of episodes of incontinence per day of 1 or less). Assessments were done by personnel blinded to the index test result. For outcome analysis, an ‘improvement’ reflected better outcome in at least one of the three objective measures, in addition to reported subjective improvement. A deterioration in any one of these clinical elements resulted in an overall outcome of ‘no improvement’. All outcome data were processed on an anonymous database.

### Statistical analysis

Sample means and variation: Samples were grouped into primary CSF collection (i.e. taken prior to the shunt) and secondary (i.e. taken to test the functioning of a shunt or as part of a revision surgery). Mean levels of T-tau and Aβ-42 were reported with standard deviation. Mean levels were compared between the various sample collection groups (lumbar vs. primary ventricular vs. secondary ventricular) using analysis of variance (ANOVA with a Geisser-Greenhouse’s epsilon correction). Linear regression analysis was performed to determine the effect of age on T-tau and Aβ-42 levels.

Exploratory analysis: ROC analysis was used to assess predictive values for overall shunt responsiveness, mobility, cognition and urinary continence. Area under the ROC (AUROC) levels were implied to suggest the predictive valve of a test as the following: 0.80–0.90 = good, 0.70–0.79 = fair, 0.60–0.69 = poor and 0.50–0.59 = no differentiation. Optimal cut-off values derived from ROC analysis are presented with 95% confidence intervals.

Pre-specified analysis: Contingency tables presenting the negative and positive predictive values (NPV and PPV), sensitivity and specificity of T-tau and AB1-42 were then determined using the following published cut-off values for INPH: T-tau protein levels < 425.7 ng/l ± 244.3, Aβ-42 > 500 ng/l and a ratio of T-tau to Aβ-42 of <1 [17]. This was repeated using the ROC calculated optimal cut-off values. All values were expressed as percentages with ‘exact’ Clopper-Pearson confidence intervals with Fisher’s exact test results. All statistical tests were performed in GraphPad Prism v6.0.

## Results

### Study profile

August 2006 to January 2016, a total of 144 CSF samples were analysed from 79 probable INPH patients: 31 females:48 males (31 F:48 M) of mean age 75.3 (R 55–94). Mean follow-up was 959 ± 657 days (mean ± SD). Figure 1 outlines the study profile.

### Demographics

The frequency of clinical characteristics in both the shunt responsive and non-responsive groups is demonstrated in Table 1. There was no significant difference in the frequency of co-morbidities (Table 1).

**Table 1:** Clinical characteristics

	Overall (*n* = 65)	Shunt responsive (*n* = 46) 70%	Non-responsive (*n* = 19) 30%	*p*-value
Mean age (years)	75.3	74.9	75.7	1.00
Alzheimer’s disease	5 (6.33%)	3 (6.62%)	2 (15.8%)	1.00
Parkinson’s disease	5 (7.69%)	4 (8.70%)	1 (5.26%)	1.00
Lewy-body dementia	3 (4.62%)	3 (6.52%)	0 (0.00%)	0.54
Frontal-temporal lobe dementia	1 (1.54%)	1 (2.18%)	0 (0.00%)	1.00
Cerebral infarct or haemorrhage	3 (4.62%)	2 (4.35%)	1 (5.26%)	0.62
Other neurological disease ^a^	4 (6.15%)	2 (4.35%)	2 (15.8%)	0.57
Depression	4 (6.15%)	1 (2.18%)	3 (10.6%)	0.07
Coronary/peripheral vascular disease	40 (61.5%)	29 (63.0%)	11 (57.9%)	0.78
Urinary dysfunction^b^	10 (15.4%)	8 (17.4%)	2 (15.8%)	0.71
Musculoskeletal disease^c^	13 (20.0%)	10 (21.7%)	3 (10.6%)	0.74
Visual disturbance	1 (1.54%)	2 (4.35%)	1 (5.26%)	1.00

^a^Included: Progressive supranuclar palsy, multiple sclerosis, motor neurone diseases, multiple system atrophy, myasthenia gravis, amyloidosis, peripheral neuropathy, spinocerebellar ataxia, benign paroxysmal positional vertigo, Meniere’s disease, essential tremor and other movement disorders

^b^Including benign prostatic hypertophy

^c^Including spinal stenosis

T-tau levels and Aβ-42 ratio levels correlated with increasing age on linear regression analysis. When the results from the three patients under the age of 60 were excluded from the analysis, this correlation was no longer significant (*p* = 0.09, R^2^ = 0.04). The three patients under the age of 60 were included in the full analysis.

The mean duration of the symptoms of INPH prior to CSF analysis was 290.5 ± 491.1 days. The mean time to VP shunt insertion after LP or LD was 226.9 ± 218.7 days. The mean time from VP shunt insertion to reservoir tap test was 739.1 ± 325.5 days.

### Neurodegenerative disease

Fourteen patients had a post-operative neurodegenerative diagnosis (5 Parkinson’s disease, 5 AD, 3 Lewy-body dementia and 1 frontal-temporal lobe dementia). The presence of a neurodegenerative diagnosis did not significantly affect outcome (Table 1). Eleven of 14 patients with a neurodegenerative diagnosis were still shunt responsive.

### Mean levels per site

Mean levels of Aβ1-42 and T-tau per sampling method are presented for INPH and INPH patients with a co-existing neurodegenerative disease (Table 2). The range of T-tau levels in primary lumbar CSF varied from 68 to 872 ng/l and from 56 to 2085 ng/l in primary ventricular samples. Aβ1-42 levels ranged from 111 to 911 ng/l in lumbar samples and 100–1231 in primary ventricular samples. Mean CSF levels of T-tau in samples taken during LP or LD were significantly lower than in the samples seen during VP shunt insertion (*p* = 0.001).

**Table 2:** Mean levels of T-tau and Aβ-42 in 144 CSF samples taken from various sites (ng/l)

	Primary (before VP shunt insertion) (*n* = 101)				Secondary (after VP shunt insertion) (*n* = 43)		
	Lumbar (*n* = 30)		Ventricular (*n* = 71)		Lumbar (*n* = 2)	Ventricular (*n* = 41)	
Sample ± SD	LP (*n* = 7)	LD (*n* = 23)	EVD (*n* = 1)	VP (*n* = 70)	Infusion (*n* = 2)	Tap test (*n* = 17)	VP revision (*n* = 24)
T-tau overall	303 ± 245	275 ± 205	903	600 ± 429	541 ± 110	373 ± 290	620 ± 664
INPH + ND^a^	462 ± 446	282 ± 170		601 ± 455	–	655 ± 755	319 ± 343
INPH^b^	223 ± 138	228 ± 159	–	522 ± 110	–	372 ± 289	538 ± 261
Aβ-42 overall	675 ± 524	534 ± 206	198	460 ± 280	532 ± 354	571 ± 300	576 ± 286
INPH + ND^a^	909 ± 849	710 ± 137	–	597 ± 455	–	425 ± 170	413 ± 216
INPH^b^	675 ± 524	525 ± 203	–	472 ± 532	–	464 ± 295	565 ± 285

^a^INPH + ND = Only patients with a diagnosis of INPH + a post-operative diagnosis of neurodegenerative diagnosis

^b^INPH = excluding results from patients with a post-operative or delayed diagnosis of neurodegenerative diagnosis

### ROC analysis of T-tau and Aβ-42 and shunt response

AUROC for lumbar CSF levels of T-tau was 0.6, for T-tau/Aβ-42 0.62, respectively, with Aβ-42 being 0.5. The AUROC for ventricular CSF levels of T-tau, Aβ-42 and T-tau/Aβ-42 were 0.70, 0.51 and 0.64, respectively.

**Fig. 2:**
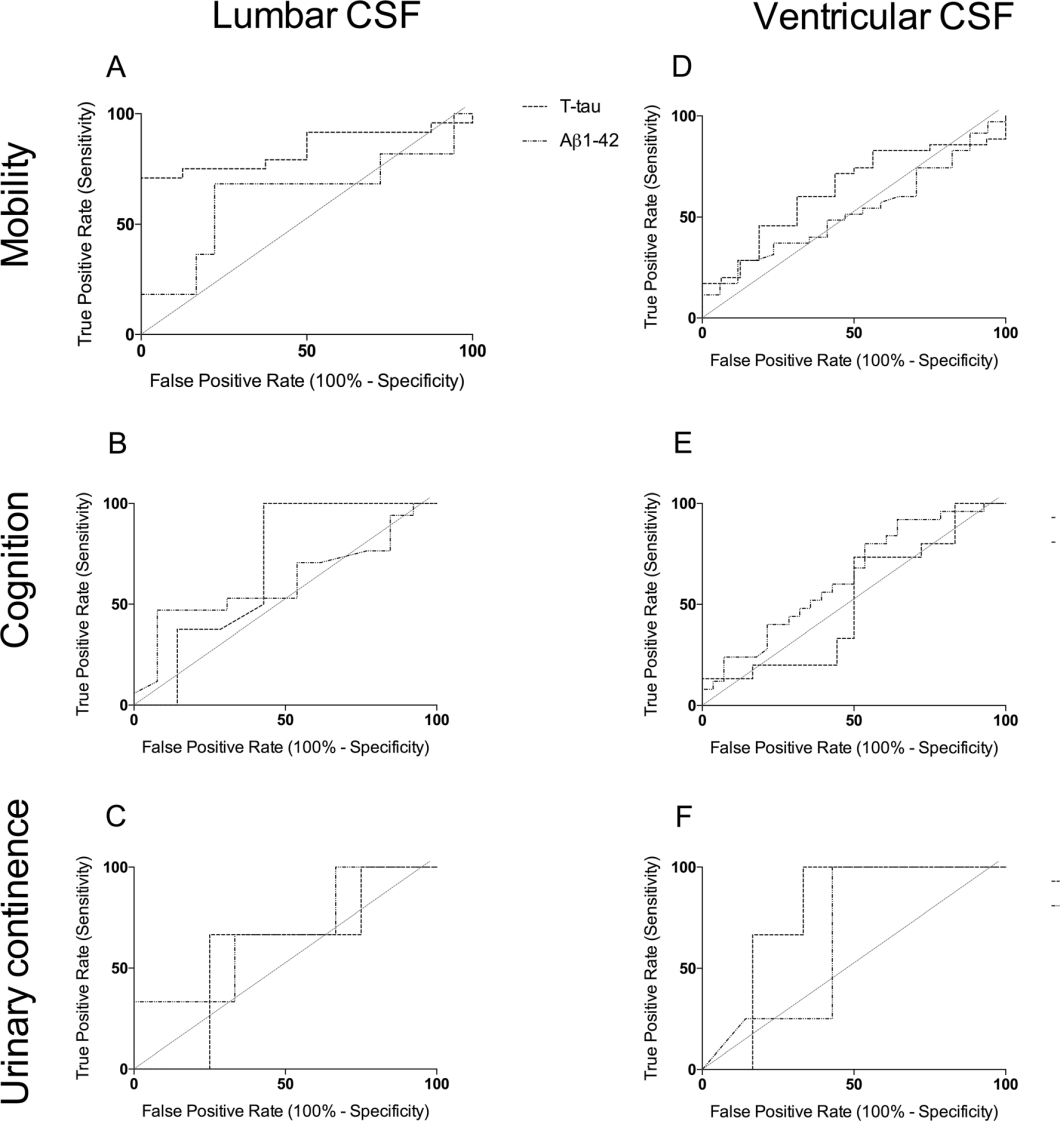
ROC curve demonstrating ability CSF levels of T-tau, Aβ1-42 levels and the ratio T-tau/Aβ1-42 to predict improvement in the triad of symptoms **a–c** lumbar primary CSF **a** mobility (*n* = 21), **b** cognition (*n* = 13) and **c** continence (*n* = 5). **d–f** Ventricular primary CSF **d** mobility (*n* = 36), **e** cognition (*n* = 17) and **f** continence (*n* = 9)

The analysis of lumbar and ventricular samples was repeated for the individual components of outcome: mobility, cognition and urinary continence. T-tau and Aβ-42 levels in both lumbar (Fig. 2a–c) and ventricular samples (Fig. 2d–f) were poor predictors of outcome, with the exception of lumbar T-tau for predicting improvement in mobility. Lumbar CSF T-tau had an AUROC of 0.84 (*p* = 0.04), making it a potentially good predictor of mobility outcome (Fig. 2). Although graphically ventricular T-tau and urinary continence outcome appears promising, with an AUROC of 0.78, the result is not significant (*p* = 0.19).

### Predictive values for T-tau and Aβ-42 and shunt response

Optimal predictive cut-off values derived from ROC analysis for T-tau in lumbar samples were < 196.5 ng/l and < 525 ng/l in ventricular samples. The predictive values of degenerative markers in lumbar CSF and ventricular CSF are presented in Tables 3 and 4, respectively.

**Table 3:** Contingency table primary lumbar CSF (LD or LP) (*n* = 30)

**T-Tau** [ROC - cut-off]	T-Tau low <425 [196.5] ng/l	Normal	Total	
Shunt responsive	17 [13]	6 [10]	23	Sensitivity 81% [86%]
Non-responsive	4 [2]	3 [5]	7	Specificity 33% [33%]
Total	21 [15]	9 [15]		
	PPV 74% [56%]	NPV 43% [43%]		
Fisher’s exact test *p* = 0.64 [*p* = 0.38, 95% CI 34–77% sensitivity and 95% CI 29–96% specificity]				
**Aβ-42**	Aβ-42 Low	Normal	Total	
Shunt responsive	11	12	23	Sensitivity 79%
Non-responsive	3	4	7	Specificity 25%
Total	14	16		
	PPV 48%	NPV 57%		

Fisher’s exact test *p* = 1.00

**Table 4:** Contingency table for primary ventricular CSF (VP shunt insertion) (*n* = 57)

T-Tau [ROC - cut-off]	T-Tau low <425 [<525] ng/l	Normal	Total	
Shunt responsive	26 [30]	12 [8]	38	Sensitivity 81% [81%]
Non-responsive	6 [7]	13 [12]	19	Specificity 52% [60%]
Total	32 [37]	25 [20]		
	PPV 68% [78%]	NPV 68% [63%]		
Fisher’s exact test *p* = 0.02 [*p* = 0.02, 95% CI 59–86% sensitivity and 95% CI 34–79% specificity]				
Aβ-42	Aβ-42 Low	Normal	Total	
Shunt responsive	23	15	38	Sensitivity 64%
Non-responsive	13	6	19	Specificity 28%
Total	36	21		
	PPV 61%	NPV 32%		

Fisher’s exact test *p* = 0.77

### Complications

One patient developed an infection as a result of a clinically indicated reservoir tap test. This patient was treated with intravenous antibiotics. There were no mortalities associated with CSF sampling.

## Discussion

### Mean T-tau and Aβ1-42 levels in the CSF of patients with probable INPH

We have performed a singe-centre analysis of T-tau and Aβ1-42 levels in the CSF of patients with probable INPH. This is one of the most comprehensive presentations of CSF samples from INPH patients to date, thus providing further reference values to the current literature [2]. The mean lumbar CSF levels of T-tau in INPH were found to be lower than levels expected in normal controls, a finding observed in several studies [8, 11, 13]. We also confirmed previous findings of a rostro-caudal gradient with significantly lower levels of T-tau in lumbar CSF than in ventricular CSF [19]. Regardless of the sampling method, the range for both markers was wide, particularly T-tau, and therefore the authors recommend caution when interpreting their levels in the context of prognosis for individual patients.

### The predictive value of Aβ1-42 and T-tau for shunt response in INPH

Ventricular Aβ1-42 and T-tau have previously been demonstrated to be potentially clinically useful biomarkers for prognosis in INPH [20]. The AUROC values for ventricular T-tau (general post-shunt outcome) and lumbar T-tau (mobility outcome) were 0.70 and 0.80. This result concurs with the findings by Kang et al. in which low levels of tau correlated with gait dysfunction [13]. Kang et al. also found that lower levels of Aβ1-42 correlated with poor cognitive outcome in INPH; however our results did not mirror this, possibly due to low numbers within this sub-group [13].

The observation that lumbar samples have a better predictive value than ventricular samples is interesting. One possible explanation may relate to the hypothesis that tau has reduced clearance in INPH, hence lower levels in CSF [8]. Therefore lumbar CSF T-tau levels may be even lower, owing to the greater distance from metabolically active tissue, thus exaggerating the predictive value.

### Patients with raised T-tau CSF can still benefit from shunt insertion

Current literature emphasises differentiating INPH and AD; however there are patients with NPH and neurodegenerative disease co-existing [8, 12, 14]. Patients with clinical signs of INPH may not be referred for neurosurgical assessment, if co-existing neurodegenerative disease is present, as it is assumed to render surgical intervention ineffective [12]. Our results show that assumption is not necessarily correct. We found that the majority of those who had neurodegenerative disease coexisting with INPH were still shunt responsive, despite this group having, on average, raised T-tau on lumbar CSF samples. We conclude raised levels of T-tau and being shunt-responsive are not mutually exclusive, and patients with raised T-tau ought not to be excluded from having a VP shunt on this criterion alone.

### Limitations

To control for the heterogeneity of our data, we analyse some of the samples in subgroups. As a result, the sub-group analysis of lumbar CSF samples (reviewing gait, cognition and continence) had reduced statistical power. The authors of this article are currently studying whether the individual CSF biomarker levels predict actual improvements (i.e. will 10% lower T-tau reflect a 20% improvement in walking speed). To get more meaningful results, greater numbers are required and therefore this is an ongoing element of this research.

### Future research

Further research into discovery of biomarkers, or even a panel of markers, in INPH remains warranted. Since this study commenced there have been advances in biomarker discovery and there are now numerous potential biomarkers to be studied in INPH [2]. One example of an effective combination of markers includes Aβ1-42, neurofilament light protein (NF-L) and phosphorylated-tau (p-Tau), a promising panel to distinguish iNPH [2]. Furthermore, there are very few studies measuring levels of marker levels over time with CSF drainage. This group is currently studying how shunt insertion changes the CSF biomarker profile in an individual. Such research may further elucidate the pathophysiology of INPH as well as assist in identifying shunt malfunctions.

## Conclusion

Raised levels of T-tau and being shunt-responsive are not mutually exclusive. This is highly relevant, since many units would have presumed such patients (i.e. those with Alzheimer’s disease and INPH) to be poor surgical candidates. In general, INPH exhibits low levels of CSF T-tau, and levels can be good predictors of outcome. However, we discourage diagnosis and outcome prediction based on T-tau levels alone, as its CSF levels are highly variable between individuals. It is likely that a combined panel of markers, including T-tau, will be a more specific method for aiding selection of patients for VP shunt insertion.
